# Exogenous DCPTA Treatment Increases Mung Bean Yield by Improving Carbon Metabolism Pathway and Up-Regulating Photosynthetic Capacity and Antioxidants

**DOI:** 10.3389/fpls.2022.796694

**Published:** 2022-04-12

**Authors:** Yuling Gao, Xiaolin Zhang, Xin Wang, Qi Zhang, Huarong Tang, Tian Qiu, HuiLai Zhang, Bingxin Zhao, Hao Wang, Xilong Liang, Yongxia Guo

**Affiliations:** ^1^College of Agriculture, Heilongjiang Bayi Agricultural University, Daqing, China; ^2^National Coarse Cereals Engineering Research Center, Daqing, China; ^3^Heilongjiang Provincial Key Laboratory of Crop Pest Interaction Biology and Ecological Control, Daqing, China; ^4^Heilongjiang Province Cultivating Collaborative Innovation Center for The Beidahuang Modern Agricultural Industry Technology, Daqing, China; ^5^Dalian Customs Technology Center, Dalian, China; ^6^Heilongjiang Plant Growth Regulator Engineering Technology Research Center, Daqing, China

**Keywords:** exogenous, DCPTA, mung bean, yield, carbon metabolism, growth and development, regulation

## Abstract

Mung bean is characterized by having a good edible and medicinal value, while its flowers and pods have low production. Being a tertiary amine, DCPTA [2-(3,4-dichlorophenoxy) triethylamine] substantially regulates the growth and development of crops, maintaining production. Yet it is still limited in terms of the regulation of DCPTA on growth and development, including the yield and sugar metabolism of mung bean. In this study, DCPTA was sprayed at the beginning of mung flowering through a two-season cultivation, to assess its effects on the yield, leaf area per plant, plant height, seed setting rate, photosynthesis, chlorophyll content, and endogenous protective enzymes. Experimental results illustrated that relative to the control (CK), the DCPTA application significantly (*p* < 0.05) improved the yield of Bailv 11 mung bean, which rose to 6.9% in 2020 and 7.8% in 2021, respectively. This effect positively corresponded to a significant (*p*<0.05) increase in the number of pods and grains per plant and pod setting rate, but a non-significant difference in 1,000-grain weight. DCPA application also increased the area and fresh weight of leaf, mung height, and its organ dry weight (i.e., leaf, branch, and stem). During plant growth over DCPTA application, the increased activities of SOD, POD, and CAT improved the net photosynthetic rate, stomatal conductance, and transpiration. In addition, transcriptome sequencing further demonstrated that DCPTA treatment significantly (*p* < 0.05) up-regulated the sucrose synthase, invertase, and fructose kinase in all organs (i.e., leaves, pod skins, and grains) of the plant. In particular, this effect was much greater in the sucrose synthesis (i.e., sucrose content) in leaves. Our study, therefore, concludes that DCPTA application promotes the yield of mung bean via likely enhancing its photosynthetic capacity and sucrose synthase, fructokinase, and beta-fructofuranosidase expression regulation.

## Background

Mung bean is an important grain legume crop in China. It is used as porridge, soup, and cake. It has a good edible value, is resistant to bacteria, has anti-tumor effects, improves immunity, reduces blood lipids, and detoxifies, with a high medicinal value (Wang et al., 2004). Despite these benefits, it has received less attention, has relatively poor management, and its production level is generally low. Improving mung bean yield without increasing the original planting area has become a critical topic, which has a significant impact on the future of mung bean planting.

Plant growth regulator refers to organic substances with molecular structures and physiological functions similar to plant hormones synthesized artificially through the chemical industry (Small and Degenhardt, 2018; Chen et al., 2021). With the development of science and technology, increasing plant growth regulators are widely used to improve crop yield and quality (Bandara and Tanino, 1995; Pal et al., 2021). α-naphthylacetic acid (NAA) promotes cell division and expansion and fruit blossom, and prevents fruit drop, therefore increasing yield (Jeong et al., 2004). Kinetin is an unnatural cytokinin to improve plant stress resistance and delays aging (Nesmith et al., 2005; Lukatkin et al., 2007). These and other growth regulators have been applied to various crops (Oshanova et al., 2021; Wang J. et al., 2021; Xu et al., 2021).

DCPTA, a new type of plant growth regulator, can be absorbed by plant stems with various beneficial effects on plant physiological processes. DCPTA, a typical representative of tertiary amine active substances, is a bioactive substance with high bioactivity and low molecular weight. It improves crop yield and quality via promoting plant protein synthesis and enzyme activity, as well as against stress resistance (Bandara and Tanino, 1995; Pal et al., 2021), such as regulating plant photosynthesis and relative enzyme activities, and the quality of agricultural products (Yokoyama et al., 1977; Xie et al., 2019a,b). For example, DCPTA treatment increases the soluble sugar content in maize seedlings and is conducive to improving dry matter accumulation and transport (Davis et al., 1986). In addition, some studies also show that DCPTA promotes the growth of different plant seedlings and increases chlorophyll content and photosynthesis, indicating that its application regulates photosynthetic responses (Gu et al., 2008). Keithly et al. (1990a) found that spraying a specific concentration of DCPTA on sugar beet leaves promoted the development of roots and leaves, significantly increasing the sucrose content. Wang et al. (2015) also found that spraying a mixture of DCPTA and CCC on maize effectively improved their chlorophyll content, reduced chlorophyll decomposition, increased the photosynthesis time, improved SOD activities in leaves and pods, and reduced damage to leaves.

Carbohydrates are the basic metabolites of plants. Sucrose is the main form of its transport, but starch is its main energy storage material. Fructose and glucose are produced after sucrose hydrolysis. Their synthesis, transport, and content in plants are affected by sucrose phosphate synthase (SPS), sucrose synthase (SuSy), acid invertase, and key enzyme activities such as invertase (SAI) (Keithly et al., 1990a,b; Gu et al., 2008). Recent studies showed that spraying different concentrations of kinetin on the leaf surface of mung bean at the beginning of flowering significantly improved photosynthetic characteristics, protective enzymes, and mung bean yield (Liu et al., 2015; Wang et al., 2015). Previous studies also found that DTA-6 treatment improved the stem transport rate of mung beans and further promoted the assimilation from “source” to “sink” (Wang N. et al., 2021). However, the effect of DCPTA on mung beans' growth, yield, and carbon metabolism is still unclear.

This study applied DCPTA to mung bean leaves at the initial flowering stage in 2020 and 2021, and deionized water was used as the control. Here, we assumed that SuSy and invertase expressions changed after DCPTA treatment via assessing the morphological indexes, photosynthetic indexes, enzyme activity changes, and transcriptional sequencing. Moreover, DCPTA treatment affected the distribution of sucrose content in different organs, improving the pod setting rate and yield. The presented research provides a theoretical basis for clarifying the role of DCPTA in mung bean production.

## Materials and Methods

### Plant Growth Conditions and DCPTA Treatment

In this study, Bailv11 [*Vigna radiata* (L.) R. Wilczek] was selected as the experimental material, provided by the National Coarse Grain Engineering Technology Research Center. Bailv11 was certified by Jilin Committee for Crop Variety Registration in 2011. It is erect, and its maturity duration is about 87 days. The plant growth regulator is 98% pure 2-(3,4-dichlorophenoxy) triethylamine (DCPTA, Shanghai Yuanye Biotechnology Co., Ltd., China).

Field trials were conducted in both 2020 and 2021 at the Heilongjiang Bayi Agricultural University, AnDa Sci-Tech Par (125°21′E, 46°24′N), Heilongjiang Province, China. The soil fertility level was determined before BaiLv11 seeding (Table 1; Supplementary Table S1). Seedlings were trimmed after emerging to maintain a total of 110,000 plants per hectare. Each replication had a plot size of 19.5 m^2^ (six rows, each 5 m long, with 0.65-m row spacing). At the beginning of flowering (about 58 days after sowing), the Bailv11 leaves were sprayed with DCPTA. Here, the control was sprayed with clean deionized water. The foliar spraying was carried out after 17:00 on a sunny day with a liquid dosage of 225 L.hm^−2^.

**Table 1:** Background data of productivity and soil fertility of the experimental fields studied from 2020 to 2021.

**Year**	**pH**	**Organic matter** ** g/kg**	**Ikali-hydro** ** N mg/kg**	**Available** ** P mg/kg**	**Available K mg/kg**	**Total** ** N g/kg**	**Total** ** P g/kg**	**Total** ** K g/kg**
2020	7.68	28.6	176.3	62.1	213	1.81	0.69	0.45
2021	7.75	28.4	172.5	61.2	208	1.78	0.65	0.43

### Sampling and Measuring Method

Mung bean samples were collected at 7, 14, 21, and 28 days after spraying. Five similar plants per replicate were selected randomly. The plants were divided into stem, leaf, and petiole. Samples were oven-dried at 80°C until a constant weight was achieved to obtain their dry weight (Yang et al., 2020).

Five similar plants per replicate were selected randomly; the plant height, stem diameter, and leaf fresh weight were measured at 7, 14, 21, and 28 days after spraying. The branch number was counted, and leaf area was measured by a leaf area meter.

Three plants per replicate were selected randomly at 1, 7, 14, 21, and 28 days after spraying. On sunny days, the measurements were performed between 9:00 and 11:00 using a portable photosynthesis system (Li-6400; LI-COR Inc., Lincoln, NE, USA). The third leaf from the top down was placed in the chamber at a photon flux density of 1,000 mol m^−2^ s^−1^, the flow rate of 500 μmol s^−1^, and 400 μmol CO_2_ mol^−1^. The net photosynthesis rate (Pn), stomatal conductance (Gs), intercellular CO_2_ concentration (Ci), and transpiration rate (Tr) were automatically recorded.

The third leaf from the top was measured and sampled at 1, 7, 14, 21, and 28 days after spraying. In one pilot area, the third leaf from the top was removed, treated with liquid nitrogen, and stored at −80°C, for the determination of Superoxide dismutase (SOD), Peroxidase (POD), Catalase (CAT), sucrose synthase (SuSy), beta-fructofuranosidase (invertase, INV) activity, and sucrose content. The pod and seed were sampled at 7, 14, 21, and 28 days after spraying, to measure the sucrose synthase, invertase activity, and sucrose content in each treatment. Fresh leaves were used to determine the chlorophyll content.

SOD, POD, and CAT were measured following previous methods (Zheng and Huystee, 1992; Thomas, 2002; Zhou et al., 2016), and sucrose synthase (SuSy) activity was assayed according to the method of Yang et al. (2018). Beta-fructofuranosidase (invertase) was measured using the method of He (1993). The sucrose content was determined following the description of Du et al. (2020).

Mung bean yield was measured at the mature stage. Each treatment included 10 plants with uniform growth harvested at 1 m height. The number of seeds, number of effective pods, and grain weight per plant, as well as 1,000-grain weight, were counted. Its theoretical yield was calculated according to the following formula:

Yield (kg hm^−2^) = number of grains per plant (units) × 1,000-grain weight (units) × number of plants per hectare (units) ÷ 1,000,000.

### RNA-Seq and qRT-PCR Analysis

Third-to-last leaves in different treatments at 7 days after spraying were taken as samples for RNA-Seq and qRT-PCR analysis. The RNA was extracted by RNAprep Pure for plant (TSP412) (Tsingke, Beijing, China), and the RNA quality was assessed by agarose gel electrophoresis using NanoDrop 2000C (Thermo, California, U.S.A). The RNA-Seq was performed by Biomarker Technologies (Beijing, China). The single-stranded cDNA was converted by HiScript II (R223, Vazyme, Nanjing, China), while the concentration and quality of cDNA were tested by NanoDrop. The primers of nine differential expressed genes (DEGs) of RNA-Seq were designed by Primer 5.0, while the Vr-actin was chosen as a reference gene (Supplementary Table S2). The qRT-PCR with three biological replicates was performed in a Light Cycler system (480II, Roche, Switzerland) with the 2×TSINGKE^®^ Master qPCR Mix. The relative expression was calculated by the formula described by (Lopez-Molina et al., 2001).

### Statistical Analysis

The test data were expressed using the mean value (SPSS 19.0, IBM, Armonk, NY, USA). The differences between the treatment mean within each measured parameter were compared via Duncan's multiple range test (*p* < 0.05).

## Results

### Effects of DCPTA on Grain Yield and Yield Components

The yield components of mung beans were characterized by 1,000-grain weight, the number of pods and seeds per plant. The pods per plant increased significantly (*p* < 0.05) in the 2 years and after DCPTA application. After spraying DCPTA, pods per plant significantly (*p* < 0.05) increased by 9.11 and 7.99% from 2020 to 2021. Compared to the control, the seeds per plant increased by 6.93 and 7.51% in 2020 and 2021, respectively. However, there was no significant difference in seeds per pod and 1,000-seed weight between DCPTA-treated and CK. Over the 2 years, the yield of mung beans treated with DCPTA was significantly higher than that of controls, and increased by 6.93 and 7.77% (Table 2). These results were in accord with increases in both the total fruit number per plant and the size of individual fruits (Keithly et al., 1990b).

**Table 2:** Effects of DCPTA on mung bean grain yield and yield components from 2020 to 2021.

**Year**	**Treatment**	**Pods per plant**	**Seed per plant**	**Seed per pod**	**1,000-seed weight (kg)**	**Yield (kg.ha** ^**−2**^ **)**
2020	DCPTA	28.38 ± 1.39a	299.21 ± 10.76a	10.54 ± 0.23a	0.044 ± 0.001a	1, 448.18 ± 12.69a
	CK	26.01 ± 1.23b	279.83 ± 11.12b	10.76 ± 0.19a	0.044 ± 0.004a	1, 354.38 ± 15.63b
2021	DCPTA	27.18 ± 1.21a	302.83 ± 11.18a	11.18 ± 0.16a	0.042 ± 0.005a	1, 399.07 ± 10.28a
	CK	25.17 ± 1.01b	281.67 ± 13.12b	11.20 ± 0.20a	0.042 ± 0.004a	1, 298.22 ± 9.26b

*The data were analyzed by ANOVA to separateits significance, Vertical bars represent mean ± SE from three independent replicates and the different letters denote significant differences (p < 0.05)*.

### Effects of DCPTA on Morphological Index and the Accumulation of Dry Matter

With the increase of spraying days of DCPTA, the plant height varied significantly compared to the control. After spraying for 14 days, the plant height of the DCPTA treatment was significantly higher than in the control. After 28 days of treatment, the plant height increased to 4.34% in 2020 and 3.60% in 2021. Dry stem weight was increased by 5.78 and 4.33% in 2020 and 2021, respectively. With DCPTA application, both leaf area per plant and leaf dry weight per plant significantly differed. Over 28 days after spraying, compared to the control, the leaf area per plant increased by 10.37 and 9.08% in 2020 and 2021, respectively. Fourteen days after spraying, the accumulation of dry matter was promoted in DCPTA-treated plants, and the difference was significant in both 2 years. The DCPTA-treated plants showed fresh and dry leaf weights that increased by 5.90 and 4.80%, and 6.64 and 6.36%, respectively, in 2020 and 2021 (Table 3).

**Table 3:** Effects of DCPTA on morphological index and the accumulation of dry matter from 2020 to 2021.

**Year**	**Days**	**Treatment**	**Plant height** **(cm)**	**Stem diameter** **(mm)**	**Number of** **branches**	**Leaf area** **(mm)**	**Total leaf** **fresh weight** **(g)**	**Dry matters** **of leaf** **(g)**	**Dry matters** **of petiole** **(g)**	**Dry matters** **of stem** **(g)**
2020	7 Day	DCPTA	38.55 ± 0.33de	6.73 ± 0.05cd	7.67 ± 0.33ab	72,993.73 ± 2,231.49ef	17.02 ± 0.17e	10.58 ± 0.30*c*de	8.45 ± 0.06c	9.24 ± 0.06de
		Control	37.75 ± 0.15e	6.43 ± 0.08d	7.00 ± 0.58b	70,378.48 ± 671.30f	17.20 ± 0.18e	10.11 ± 0.17e	8.65 ± 0.08c	9.15 ± 0.05e
	14 Day	DCPTA	40.15 ± 0.21bc	7.31 ± 0.09ab	8.33 ± 0.33a	91,624.08 ± 4,244.16bc	19.13 ± 0.26d	10.82 ± 0.22*c*de	8.56 ± 0.15c	9.98 ± 0.12bc
		Control	39.40 ± 0.66cd	7.12 ± 0.10bc	7.67 ± 0.33ab	79,005.09 ± 1,941.03de	18.23 ± 0.03de	10.29 ± 0.60de	8.96 ± 0.40bc	9.40 ± 0.03d
	21 Day	DCPTA	40.97 ± 0.64b	7.45 ± 0.23ab	8.67 ± 0.33a	95,624.08 ± 1,978.20b	20.64 ± 0.16c	11.34 ± 0.10bc	9.49 ± 0.14ab	10.14 ± 0.06b
		Control	39.58 ± 0.33cd	7.69 ± 0.08a	8.33 ± 0.33a	86,005.12 ± 2,043.63cd	19.59 ± 0.48cd	11.14 ± 0.17*b*cd	9.44 ± 0.16ab	9.81 ± 0.03c
	28 Day	DCPTA	42.77 ± 0.14a	7.66 ± 0.20a	8.33 ± 0.33a	104,619.27 ± 2,973.16a	22.45 ± 0.31a	12.67 ± 0.22a	9.77 ± 0.16a	10.61 ± 0.03a
		Control	40.99 ± 0.15b	7.39 ± 0.13ab	8.00 ± 0.00ab	94,785.53 ± 1,542.1b	21.50 ± 0.14b	12.05 ± 0.30ab	9.37 ± 0.16ab	10.03 ± 0.06b
2021	7 Day	DCPTA	36.51 ± 0.97cd	6.71 ± 0.15ab	7.67 ± 0.33ab	70,983.62 ± 1,231.49ef	16.64 ± 0.20de	8.59 ± 0.30de	7.48 ± 0.11c	9.04 ± 0.16de
		Control	35.54 ± 0.99cd	6.57 ± 0.42ab	6.80 ± 0.20b	70,125.36 ± 986.30*e*f	16.26 ± 0.31de	8.27 ± 0.17de	7.15 ± 0.09c	8.95 ± 0.20de
	14 Day	DCPTA	38.20 ± 1.23c	7.31 ± 0.76a	7.20 ± 0.20b	89,624.45 ± 2,036.17bc	17.53 ± 0.18bc	9.15 ± 0.29d	7.55 ± 0.09bc	9.27 ± 0.27bc
		Control	38.55 ± 0.33de	7.17 ± 0.18a	7.20 ± 0.20b	75,005.23 ± 3,041.14de	17.02 ± 0.17c	8.55 ± 0.16de	7.42 ± 0.09bc	9.17 ± 0.02cd
	21 Day	DCPTA	40.52 ± 1.69ab	7.76 ± 0.48a	7.80 ± 0.20ab	93,278.01 ± 2,038.29b	18.53 ± 0.80ab	10.26 ± 0.14bc	8.04 ± 0.15bc	9.68 ± 0.02abc
		Control	39.15 ± 1.21bc	7.29 ± 0.57a	7.67 ± 0.33ab	86,005.12 ± 2,043.63cd	17.46 ± 0.45bc	8.91 ± 0.10d	7.89 ± 0.06bc	9.21 ± 0.25bcd
	28 Day	DCPTA	43.12 ± 0.91a	7.80 ± 0.74a	8.20 ± 0.20a	99,619.21 ± 3,025.16a	19.45 ± 0.88a	11.37 ± 0.18a	8.62 ± 0.37a	9.88 ± 0.20a
		Control	41.62 ± 0.32b	7.60 ± 0.06a	8.20 ± 0.20a	91,325.47 ± 1,789.2b	18.56 ± 0.31ab	10.69 ± 0.02b	8.45 ± 0.19ab	9.47 ± 0.19ab

*The data were analyzed by ANOVA to separateits significance, vertical bars represent mean ± SE from three independent replicates and the different letters denote significant differences (p < 0.05)*.

### Effects of DCPTA on Pn, Tr, Gs, and Ci in Leaves

**Figure 1:**
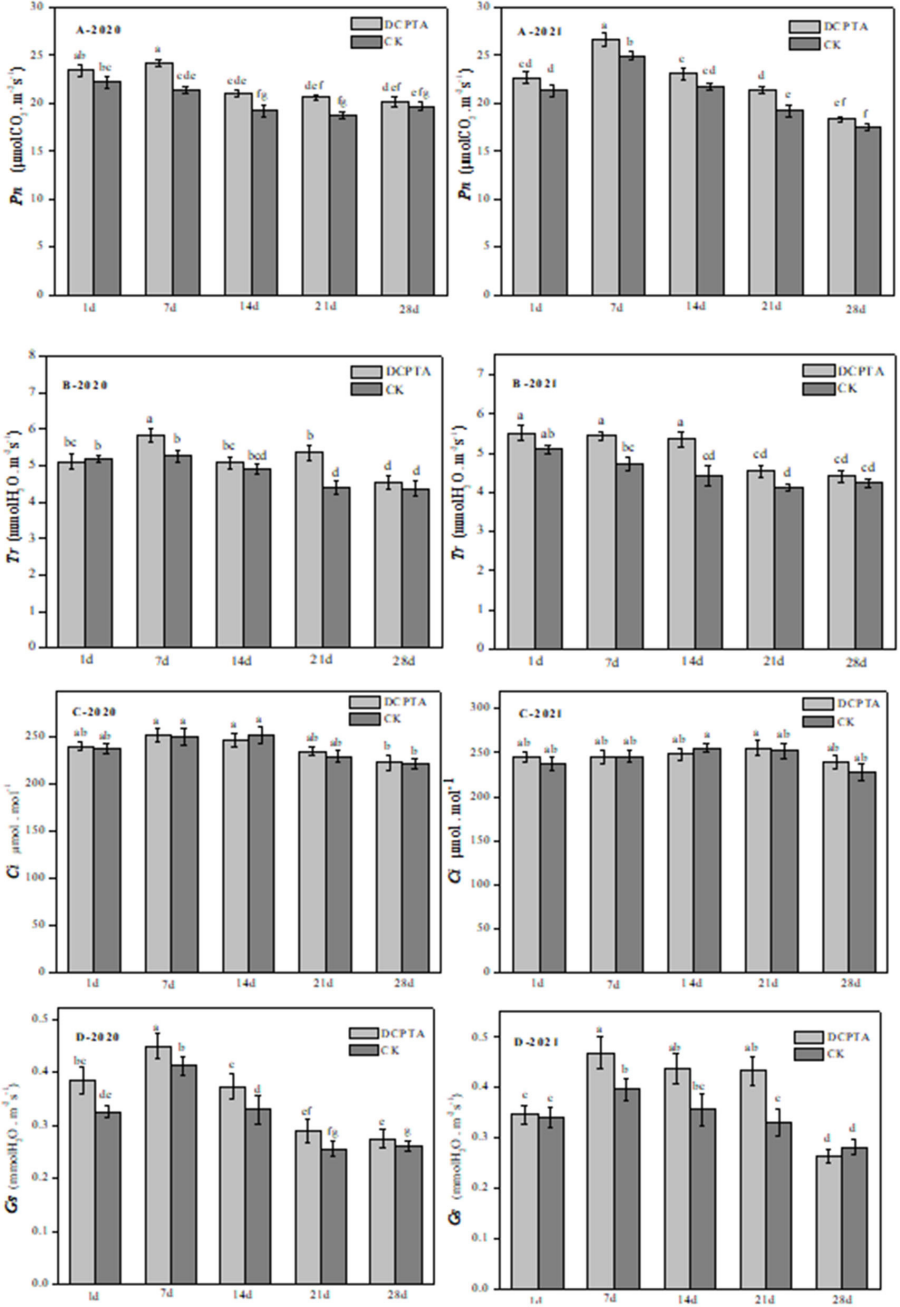
The effects of DCPTA on Pn, Tr, Gs, and Ci in leaves from 2020 to 2021. **(A)** Pn; **(B)** Tr; **(C)** Ci; and **(D)** Gs at 1–28 days after spraying. Data were analyzed by ANOVA to separate significance. Vertical bars represent mean ± SE from three independent replicates and the different letters denote significant differences (*p* < 0.05).

After DCPTA treatment in different growth stages, except for 1 and 28 days, Pn was changed significantly at all time points (Figure 1). Compared to the control, after being sprayed with DCPTA for 7 days, Net photosynthesis (Pn) significantly increased by 13.06 and 9.04% from 2020 to 2021 (Figure 1A), respectively. Transpiration rates (Tr) were similarly increased by 10.69 and 15.13% at 7 days after spraying from 2020 to 2021 (Figure 1B), respectively. From 7–21 days, stomatal conductance (Gs) was significantly different, with an increase of 9.76, 12.12, and 16.00% in 2020 (Figure 1D-2020), and 17.50, 22.22, and 30.30% in 2021 (Figure 1D-2021). However, for intercellular CO 2 concentration (Ci), there was no significant change in either 2020 or 2021 (Figure 1C).

### Effects of DCPTA on Chlorophyll Content in Leaves

**Figure 2:**
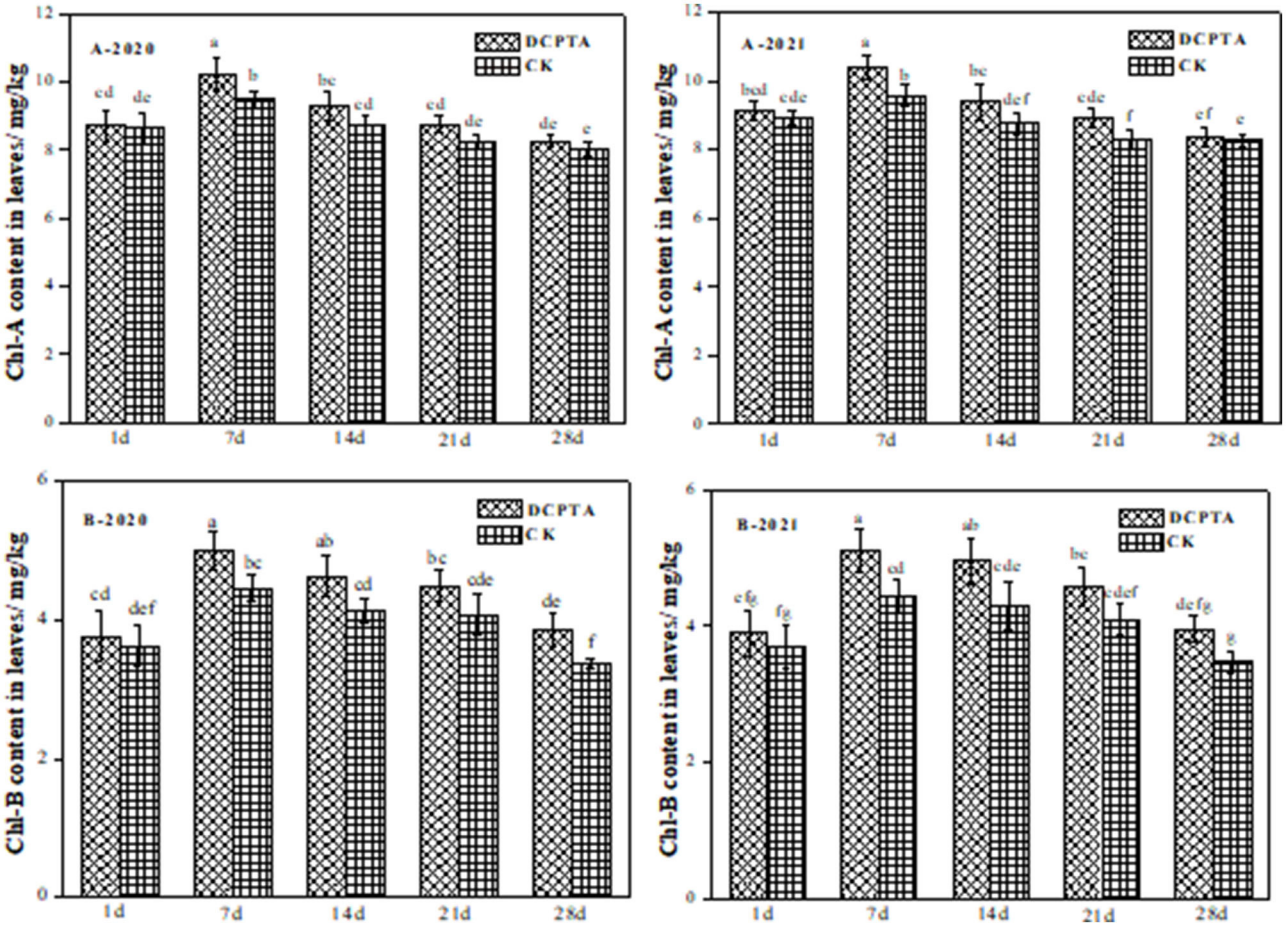
The effect of Chl-A and Chl-B in leaves on days after spraying from 2020 to 2021. **(A)** Chl a content; and **(B)** Chl b content of leaves at 1–28 days after spraying in 2020 and 2021. Data were analyzed by ANOVA to separate significance. Vertical bars represent mean ± SE from three independent replicates and the different letters denote significant differences (*p* < 0.05).

Chlorophyll is the main photosynthetic pigment, and its content represents photosynthetic capacity. Chlorophyll content was significantly dependent on year, DCPTA, and sampling time. With growth time, both chlorophyll a and chlorophyll b contents increased firstly and then decreased (Figure 2). For chlorophyll a, DCPTA treatment was not significantly different at 1 day after spraying. Yet at 7 days after DCPTA spraying, chlorophyll a content increased by 7.93 and 8.54%, 2020 to 2021 (Figure 2A). Compared to the control, the chlorophyll b content at 7 days after DCPTA application increased significantly by 12.10 and 15.05% from 2020 to 2021, respectively (Figure 2B). DCPTA treatment showed the best effect, and the total chlorophyll content increased.

### Effects of DCPTA on SOD, POD, and CAT in Leaves

**Figure 3:**
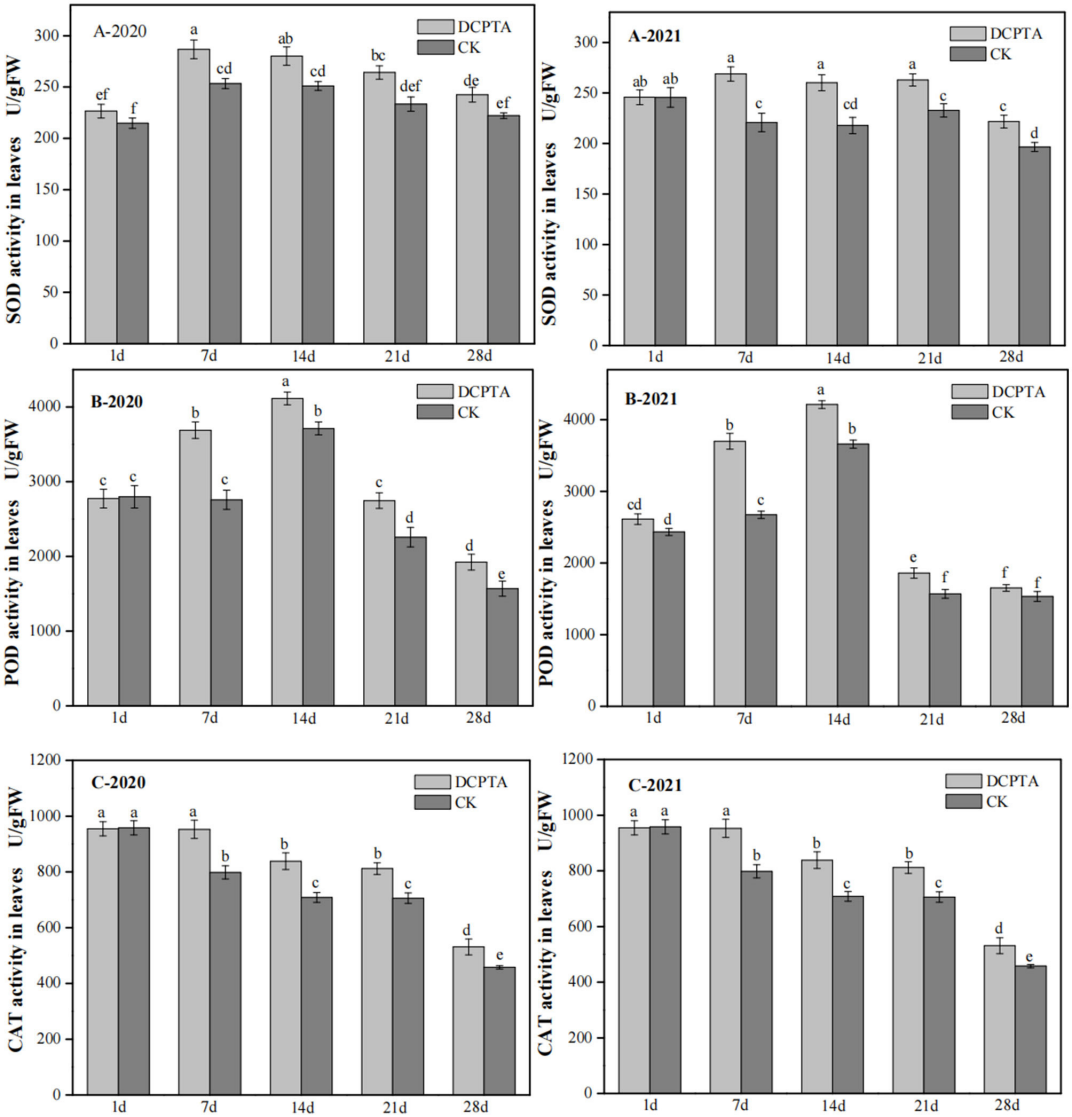
The effect of SOD, POD, and CAT activity in leaves on days after spraying from 2020 to 2021. **(A)** SOD activity; **(B)** POD activity; and **(C)** CAT activity in leaves at 1–28 days after spraying in 2020 and 2021. Data were analyzed by ANOVA to separate significance. Vertical bars represent mean ± SE from three independent replicates and the different letters denote significant differences (*p* < 0.05).

Endogenous protective enzyme activity represents anti-aging ability, and a higher activity can delay leaf senescence. Superoxide dismutase (SOD) is an important antioxidant to catalyze the removal of superoxide free radicals and protect cells from oxidative damage (Feng et al., 2015; Islam et al., 2021). From 1 to 28 days after spraying, the SOD activity showed an upward trend and a downward trend after that from 2020 to 2021 (Figure 3). The SOD activity of Bailv11 treated with DCPTA was higher than that of the control, except for the first day, and reached a peak 7 days after spraying. DCPTA could slow the rate of decline in SOD activity, and its effect was significant. Compared to the control, after spraying in 2020, the SOD activity in plants was increased by 13.14, 11.59, and 13.21% at 7, 14, and 21 days, respectively (Figure 3A). The SOD activity in 2021 was similar to 2020, and the DCPTA showed the best effect for increasing SOD activity than the control. There was no significant difference between DCPTA and control 1 day after spraying.

Peroxidase (POD) widely exists in plants. It can protect chloroplasts, participate in the production of reactive oxygen species, and trigger membrane lipid peroxidation (Bolwell and Wojtaszek, 1997). POD activity increased first and then decreased in both treatment and control (Figure 3B). The POD activity increased from 7 to 28 days after spraying in 2020 and appeared to show a significant difference (Figure 3B-2020). In 2021, compared to the control, 28 days after spraying, this difference was not significant (Figure 3B-2021). It may be caused by different years and climatic conditions, as there was no significant difference between the other dates of the 2 years. The POD activity increased by 33.76 and 21.76% at 7 days after spraying from 2020 to 2021. In general, DCPTA treatment improved POD activity.

Catalase (CAT) in all plant cells can decompose high concentrations of H_2_O_2_, which was produced in plants, and eliminate the toxicity of reactive oxygen species (Zhang and Kirkham, 1994). The CAT activity had no significant change at 1 day after spraying over the 2 years (Figure 3C) but showed an increase at 7 days after spraying in all periods, compared to the control. From 7 to 28 days, the CAT activity was increased by 19.35, 18.35, 15.02, and 16.03% in 2020 (Figure 3C-2020), and increased by 20.19, 18.85, 13.87, and 14.05% in 2021, respectively (Figure 3C-2021). DCPTA-treated plants showed the best result for delaying decreased activity of CAT.

### RNA-Seq Analysis

Raw RNA-seq data showed that total reads were from 49.86 million to 64.64 million, while the Q20 and Q30 values for all raw data were higher than 97 and 93% (Supplementary Table S2), proving that the quality of RNA-Seq could be used for further analysis.

The data have been uploaded to NCBI, with the accession number PRJNA717930 (Supplementary Table S3).

**Figure 4:**
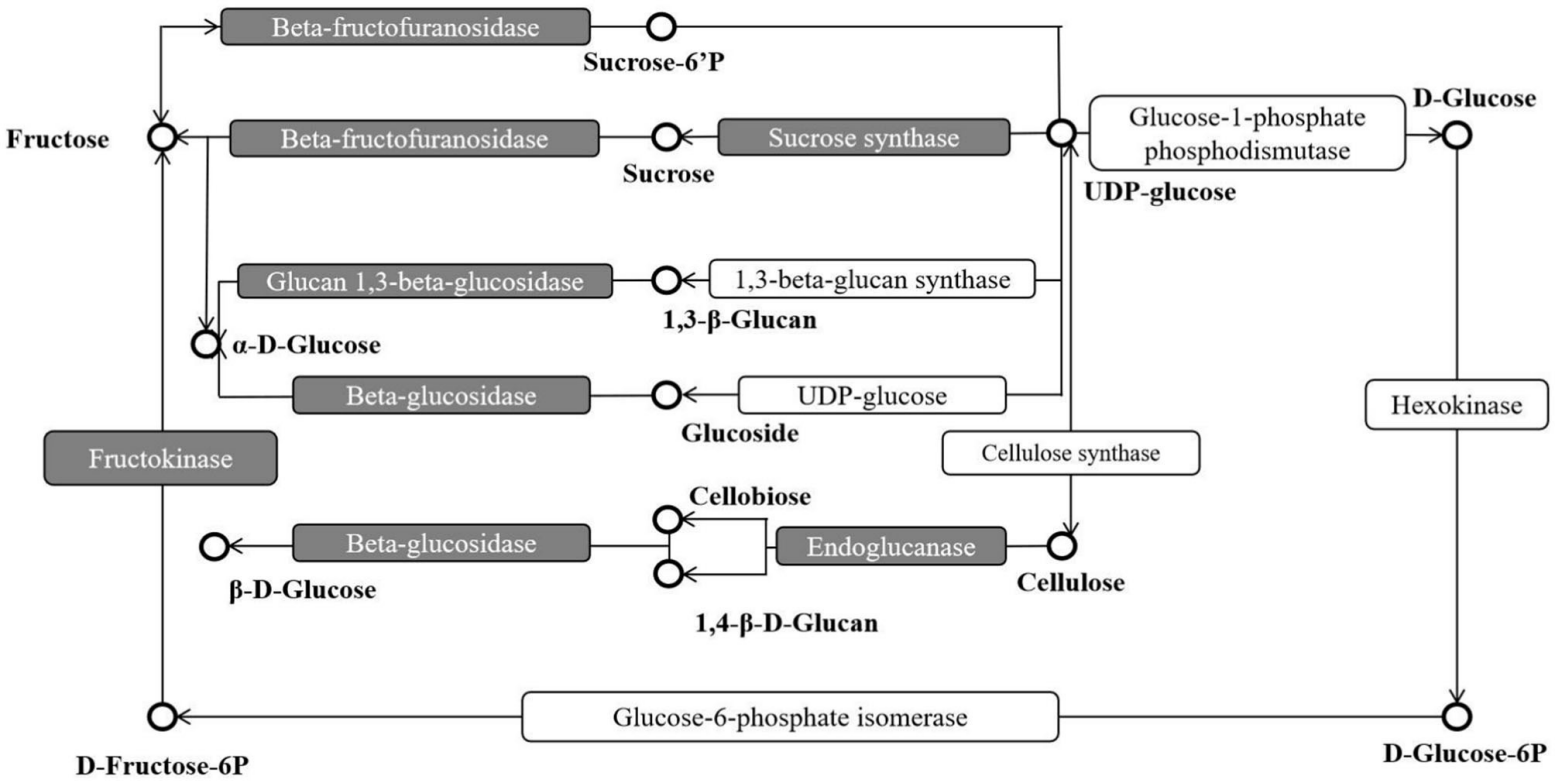
The DEG-enriched points in the KEGG pathway “Starch and Sucrose metabolism”. The sucrose synthase, fructokinase, and beta-fructofuranosidase in red were DEG-enriched points and they might eventually affect sucrose.

Compared with two treatments, 1,064 DEGs were revealed, including 269 up- and 795 down-regulated. These DEGs were used as candidate genes for further analysis, such as GO and KEGG. In GO analysis, the DEGs were enriched in biological regulation, growth, and nutrient reservoir activity, indicating that these terms might respond to the DCPTA to regulate the yield. In KEGG analysis, the *q*-value of three pathways [i.e., starch and sucrose metabolism (ko00500), plant-pathogen interaction (ko04626), and MAPK signaling pathway-plant (ko04016)] were <0.05, which significantly altered pathways and candidate pathways in this study (Figure 4).

### Starch and Sucrose Metabolism Pathway Analysis

**Figure 5:**
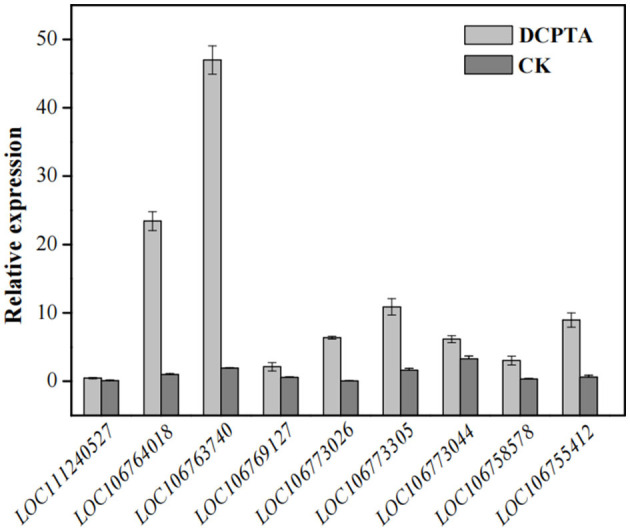
The expression of the DEGs enriched in sucrose synthase, fructokinase, and beta-fructofuranosidase paths. *LOC111240527, LOC106764018, and LOC106763740* of DEGs enriched in beta-fructofuranosidase; *LOC106769127, LOC106773026, LOC106773305, LOC106773044*, and *LOC106758578* enriched in sucrose synthase, *LOC106755412* enriched in fructokinase.

In the starch and sucrose metabolism (ko00500) pathway, the DEGs were enriched in sucrose synthase, fructokinase, and *beta-fructofuranosidase*, indicating that exogenous DCPTA might regulate this pathway to increase the yield, especially for DEGs. The expression of some DEGs had been tested by *q*RT-PCR, in which the DEGs are enriched in the sucrose synthase, fructokinase, and beta-fructofuranosidase (invertase). The result of the qRT-PCR showed that the expression of DEGs enriched in the sucrose synthase, fructokinase, and *beta-fructofuranosidase* were significantly different to the RNA-Seq analyzed, verifying that starch and sucrose metabolism pathway was a candidate pathway in responding DCPTA to increase the yield of mung bean (Figure 5). In addition, the activities of the sucrose synthase, fructokinase, and *beta-fructofuranosidase* in the pathway were determined.

**Figure 6:**
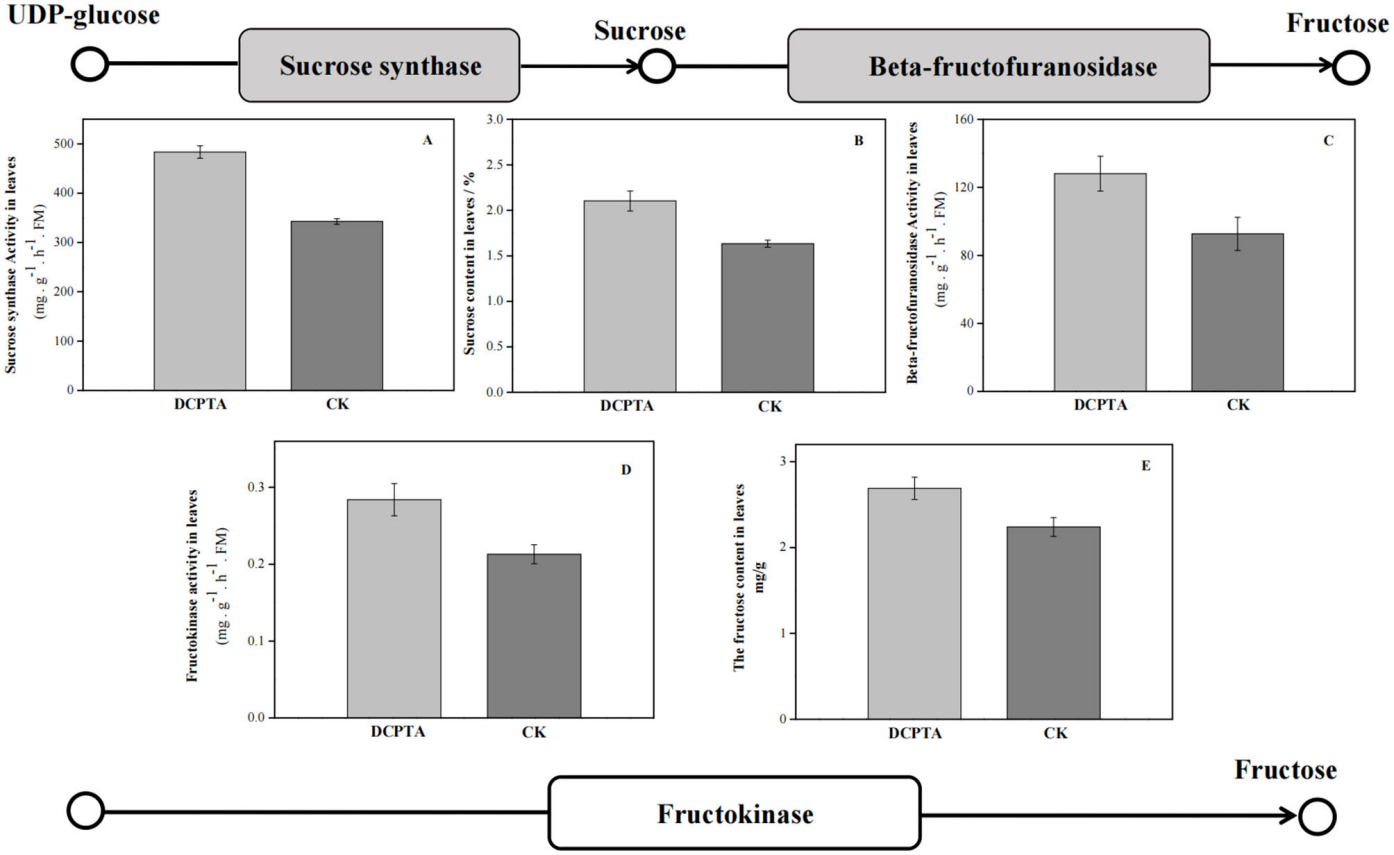
KEGG-enrichment analyses of DEGs. **(A)** Sucrose synthase activity; **(B)** Sucrose content; **(C)** Beta-fructofuranosidase activity; **(D)** Fructokinase activity; and **(E)** Fructose content at 7 days after spraying.

The physiological index further proved that the starch and sucrose metabolism (ko00500) pathway responded to exogenous DCPTA. At 7 days after spraying, both SuSy and sucrose content increased (Figure 6). Compared to the control, with the increase of beta-fructofuranosidase, the fructose content in leaves was significantly higher, and the sucrose and fructose contents increased by 28.78 and 20.09%, respectively. Fructokinase activity and fructose content were significantly higher than in the control.

### Effects of DCPTA on Sucrose Content in Leaves, Pods, and Grain

**Figure 7:**
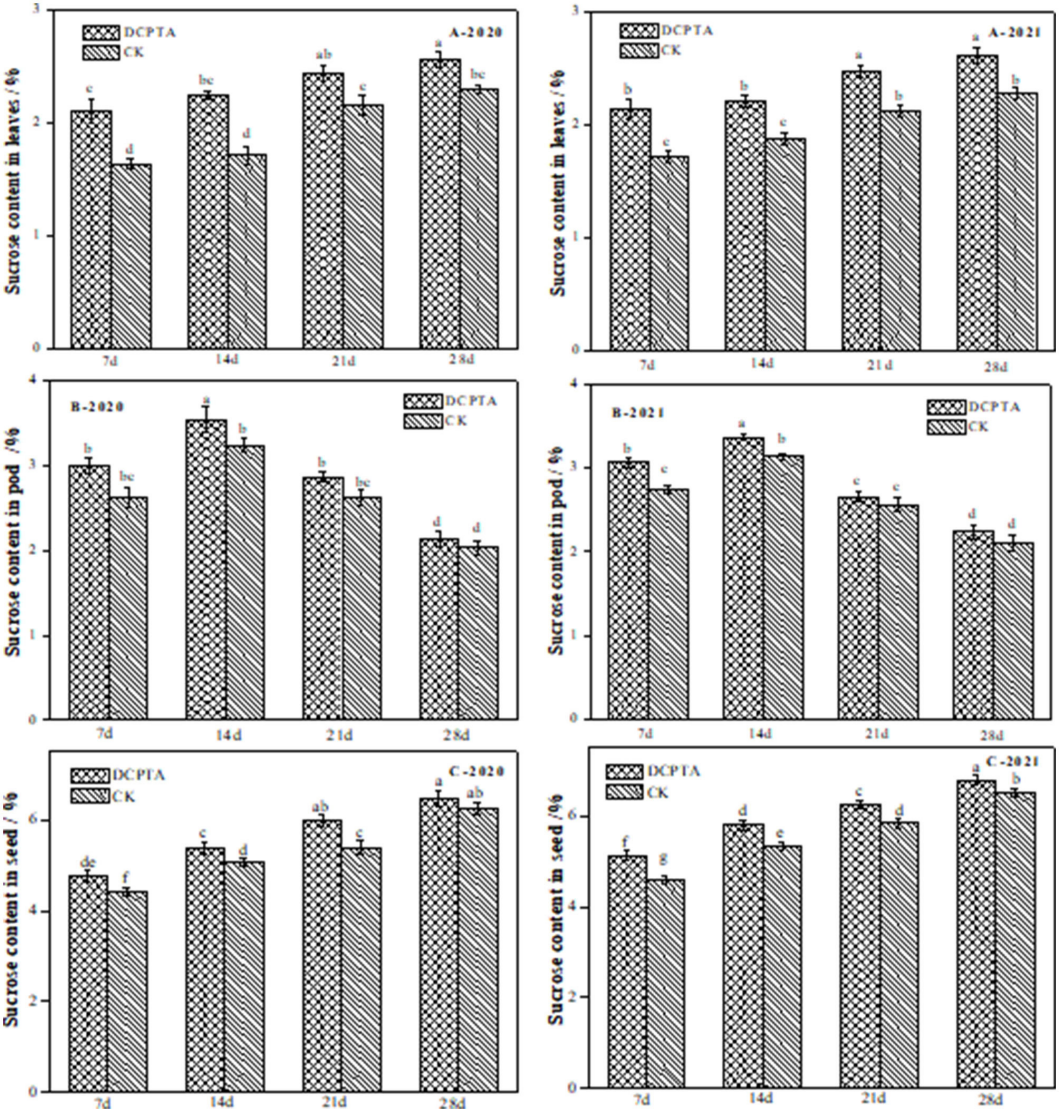
Effects of DCPTA on sucrose content in leaves, pods, and grain from 2020 to 2021. **(A)** Sucrose content in leaves; **(B)** Sucrose content in pods; **(C)** Sucrose content in grain at 7–28 days after spraying in 2020 and 2021. Data were analyzed by ANOVA to separate significance. Vertical bars represent mean ± SE from three independent replicates and the different letters denote significant differences (*p* < 0.05).

The sucrose content in leaves reflects the supply capacity of assimilates in “source” organs (Liu and Li, 1996). The sucrose content in leaves increased slowly (Figure 2). Significant differences in sucrose content between the DCPTA and control treatments were obtained (*p* < 0.05) in Bailv11 leaves from 7 to 28 days (Figure 7A). Compared to the control, the sucrose content in leaves from 7 to 28 days increased by 1.10, 1.29, 1.31, 1.13, and 1.11-fold in 2020 (Figure 7A-2020), and by 1.25, 1.18, 1.17, and 1.15-fold in 2021 (Figure 7A-2021), respectively. Compared to the control, the increase rate was the greatest for DCPTA at 7 days over 2 years.

The sucrose content in pods was significantly higher than that of the control from 7 to 14 days, increased by 14.56 and 9.26% in 2020 (Figure 7B-2020), and by 12.20 and 7.34% in 2021 (Figure 7B-2021). However, from 21 to 28 days, there was no significant difference compared to the control over 2 years.

Sucrose content in the grain of Bailv11 rapidly increased from 7 to 28 days over 2 years (Figure 7C). The sucrose content in DCPTA-treated samples was higher than in controls at all time points from 2020 to 2021. The sucrose content in the grain of Bailv11 was highest at 28 days. Significant differences in sucrose content between DCPTA and control treatments were obtained (*p* < 0.05) for both varieties from 7 to 21 days after spraying.

### Effects of DCPTA on Sucrose Synthase and Beta-Fructofuranosidase in Leaves, Pods, and Grain

**Figure 8:**
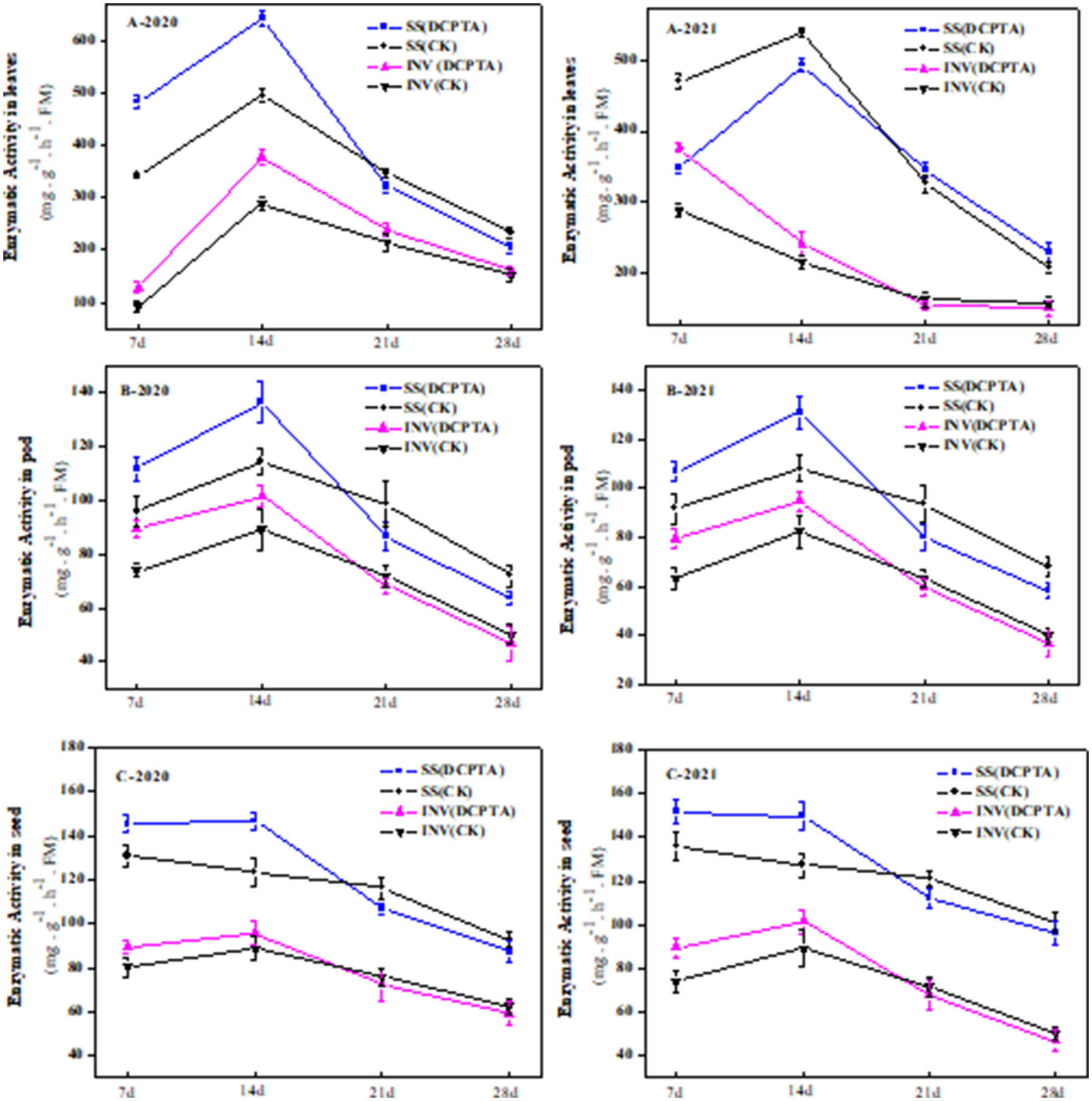
Effects of DCPTA on sucrose synthase and beta-fructofuranosidase in leaves, pods, and grain from 2020 to 2021. **(A)** Sucrose synthase and beta-fructofuranosidase activity in leaves; **(B)** Sucrose synthase and beta-fructofuranosidase activity in pods; and **(C)** Sucrose synthase and beta-fructofuranosidase activity in grain at 7–28 days after spraying in 2020 and 2021. Data were analyzed by ANOVA to separate significance. Vertical bars represent mean ± SE from three independent replicates and the different letters denote significant differences (*p* < 0.05).

In sucrose transport, the enzymes involved in sucrose metabolism mainly include invertase and sucrose synthase. At 7 and 14 days after spraying, the SuSy and beta-fructofuranosidase (invertase) activities in leaves were significantly higher than in the control, and sucrose synthase was increased by 41.06 and 29.74%, respectively, in 2020 (Figure 8A-2020), and by 42.15 and 14.71% in 2021 (Figure 8A-2021), respectively. Invertase activities were increased by 38.19 and 30.69% at 7 and 14 days in 2020 (Figure 8A-2020), and by 31.11 and 12.04% in 2021 (Figure 8A-2021), respectively. From 14 to 28 days after spraying, both DCPTA treatment and the control showed a decreasing trend. The DCPTA-treatment was more conducive to the accumulation of sucrose content in the leaves.

In both DCPTA treatment and control, the invertase activity of pods was increased first and then decreased. The invertase activity reached the maximum 14 days after spraying and was 12.22 and 12.90% higher from 2020 to 2021 than the control (Figure 8B). From 21 to 28 days, the invertase activity was slightly lower than the control over the 2 years.

From 7 to 28, the SuSy activity in the pod of Bailv11 showed a single peak trend of increase and decrease. From 7 to 14 days, compared to the control, the SuSy activity of DCPTA-treated was significantly higher. In both DCPTA and control treatments, the SuSy activity reached the maximum at 14 days after spraying and increased by 15.92 and 17.34% from 2020 to 2021, respectively. With the organ maturity from 21 to 28 days after spraying, the SuSy and invertase activity in the pod was less and less, being conducive to the assimilate accumulation to meet the needs of grain growth and development.

At 7 and 14 days after spraying, the invertase activity of the grain was significantly higher than that of the control, which increased by 11.67 and 7.94% in 2020 (Figure 8C-2020), and 21.29 and 13.92% in 2021 (Figure 8C-2021). At 21 and 28 days after praying, compared to the control, the invertase activity was decreased. Compared to the control, the SuSy in seed from 7 to 14 days was similar in invertase and increased by 11.71 and 17.33% in 2020, and 11.23 and 18.64% in 2021. From 21 to 28 days, DCPTA treatment decreased sharply, even slightly lower than the control. The change of invertase activity coincided with the change of grain sucrose content.

## Discussion

In this study, DCPTA treatment significantly improved the pod setting rate and yield. The drop of pod setting rate induced by falling flowers is one of the main reasons for the low yields of mung beans. After DCPTA treatment, flower drop was reduced, but the pod setting rate increased, thus increasing the yield. The actual yield in 2 years was higher than the control. For example, the actual yield increased by 6.93% in 2020 and by 7.77% in 2021, proving that DCPTA significantly promotes its production. These results are in accord with the increase of both the total fruit number per plant and the size of individual fruits (Keithly et al., 1990b). In this study, after spraying DCPTA at the beginning of flowering, there was no significant difference in stem diameter and the branch numbers between the treatment and the control. However, the plant height, leaf area, total leaf fresh weight, leaf dry weight, and stem dry weight were significantly higher than the control in the growth process. This result is consistent with a previous study that examined that DCPTA can increase corn yield and leaf area (Liu et al., 2018; Wang et al., 2018; Li et al., 2019). There were two potential reasons for these results. Firstly, DCPTA promotes the generation of cytokinin by regulating the biological activity of mevalonic acid (Li et al., 2016). Cytokinin promotes cell division, and this effect may be the potential reason that the leaf area and plant height increased after applying DCPTA. Secondly, It may be due to the increase of chlorophyll content, and then Pn, Gs, Tr were increased in leaves, improving the assimilation of CO_2_ and the production of photosynthetic compounds (Nakamura and Nakamura, 2016). Current research also showed that DCPTA treatment enhanced the photosynthesis of maize seedlings. Indeed, it was suggested that DCPTA increased the photosynthetic rate by raising the content of chlorophyll, sugar, and starch (Richter et al., 1987; Gu et al., 2014).

Larger Gs was conducive to the entry of CO_2_ into the plant via maintaining a larger Tr, reducing the resistance of leaf epidermis, conducive to photosynthesis. This study has shown that under non-stress conditions, DCPTA treatment promotes the growth of mung beans, being consistent with the previous research results on soybeans, radish, cotton, sugar beet, and tomato (Wang et al., 2016). Under non-stress conditions, DCPTA treatment can improve photosynthetic capacity, which is consistent with the previous research results on tomatoes (Keithly et al., 1990b).

Carbon metabolism refers to a series of physiological and biochemical processes in plants, assimilating CO_2_ into organic carbohydrates during photosynthesis and dissimilating organic carbon into carbon dioxide during respiration and photorespiration, which includes the synthesis, degradation, and transformation of photosynthetic product sucrose. Exogenous application alters carbon metabolism, yield, and the quality of crops. Carbon metabolism is a crucial method of crop material transformation. Sugar is the basic constituent of plants, which affects the patterns and mechanisms of crop material metabolism, energy transformation, growth, and development. SuSy is one of the important enzymes for controlling the transport of carbon skeleton to carbon metabolism, promoting sucrose into various metabolic pathways, improving production quality, and increasing yield (Lu et al., 2005; Cline et al., 2018). Previous studies have shown that applying appropriate concentrations of DCPTA can improve the plant height, root length, aboveground dry weight, root dry weight, total dry weight of plants. At the same time, DCPTA can improve the photosynthetic rate by increasing PSII activity and chlorophyll, sugar, and starch contents (Gu et al., 2014). In this study, with the application of DCPTA, both chlorophyll a and chlorophyll b were higher than the control during most of the growth processes. Previous studies have proved that the application of DCPTA can improve the chlorophyll content in plants (Keithly et al., 1991). This effect is connected to DCPTA treatment, which aimed to protect photosynthetic proteins from the damage of reactive oxygen species. DCPTA treatment can increase the contents of chlorophyl a, chlorophyl b, and chlorophyl a + b in Maize seedlings and leaves under non-stress conditions (Keithly et al., 1991).

DCPTA can improve plant growth and drought resistance by regulating some enzyme activities (Xie et al., 2019b). SOD is a key active oxygen scavenger, but POD can degrade H_2_O_2_ by providing some stress resistance (Martinez et al., 2000; Lawrence et al., 2009). CAT is also the main H_2_O_2_ scavenging enzyme in plants, protecting plants from oxidative damage (Takahiro et al., 1996; Nobuhiro and Ron, 2006). There was no significant difference at 1 day after spraying compared to the control, while SOD, POD, and CAT activities were higher than the control in other periods. Overall analysis, the application of DCPTA can improve the activity of endogenous protective enzymes to improve the stress resistance of plants to resist environmental damage, which is one of the factors affecting the growth and yield of mung beans.

Sucrose is the main product of CO_2_ fixation in photosynthesis. It participates in carbon metabolism and transport, playing an important role in plant development via improving plant tolerance to various stresses (Ruan et al., 2012). During the growth and development of mung bean, sucrose is one of the initial products of photosynthesis. The content of sucrose in various organs of the plant reflect the plant's ability to synthesize, transport, and transform photosynthetic products, that is, coordinate “source,” “flow,” and “sink” (Wu et al., 2012; Gao et al., 2021). Previous studies have shown that both leaves and fruits with high sugar content do not easily fall off. In this study, after DCPTA treatment, the sucrose content in leaves was higher than that of the control during the whole growth process. This may be the reason for a reduction in flowers falling, increasing pod setting rate and yield. These experimental results were consistent with previous reports, which found that the sugar content in flowers and pods was negatively correlated with flower and pod fall (Zhang, 2004). The sucrose content in the pod first increased and then decreased, but the sucrose content in the grain increased gradually. The sucrose content in the pod from 7 to 14 days was significantly higher than in the control, but the sucrose content decreased after 21 days. At the beginning of the pod, DCPTA promoted the accumulation of sucrose in the pod, as it is conducive to the unloading of assimilates and the formation of yield. The content of grain sucrose showed an upward trend in different periods. Compared with the control, it was significantly higher at 7–21 days than that of control, but this variation was not significant at 28 days. The regulation of DCPTA was still evident in the accumulation of sucrose content. The SuSy and invertase in leaves, pod, and grains were measured. It was found that both the invertase and SuSy activity in leaves, pod skins, and grains increased first and then decreased. The two enzyme activities were closely related to sucrose metabolism, jointly regulating the production of sucrose. SuSy also plays a key role in the glucose metabolism pathway. Sucrose can be decomposed into fructose and glucose. In the early stage of grain formation, all invertase, fructokinase, and SuSy activity in leaves were higher than in the control, which was conducive to sucrose accumulation in the early stage, while these activities in pod and grain were also higher than that of the control. They decompose sucrose at the sink end to form a sucrose concentration gradient from source to sink, which provides pressure for the transportation of sucrose from phloem to fruit and ensures the continuous supply of sucrose to the sink. After 21 days after spraying, with the maturity of organs, the invertase activity in the pod decreased gradually, being more conducive to the accumulation of assimilates to meet the needs of grain growth and development. The two enzymes decreased sharply, and the change of grain invertase activity at 28 days after spraying corresponded to the change of grain sucrose content. Overall, after spraying DCPTA, all sucrose synthase, fructose kinase, and invertase-related gene expression were changed, improving the SuSy and invertase activities in plants. Enzyme activities mainly affect sucrose metabolism, and sucrose synthesis also promotes the whole carbon metabolism cycle.

Finally, our findings conclude that exogenous DCPTA can regulate crop photosynthesis, increase dry matter, and affect sugar metabolism to improve crop yield. They also indicate that exogenous DCPTA effectively improves the physiological indexes of mung beans. Transcriptome further suggests that mung bean glucose metabolism has a good response to exogenous DCPTA, affecting the yield.

## Conclusion

In this study, spraying 40 mg L^−1^ DCPTA on mung bean at the beginning of flowering significantly increased the dry matter and yield of the mung bean via improving its morphological indexes. Moreover, all activities of SOD, POD, and CAT enzymes were enhanced, being conducive to the growth and development of crops. In addition, after spraying DCPTA, all the leaf area, chlorophyll content, and photosynthetic rate increased significantly (*p* < 0.05). The products of plant photosynthesis were mainly transported to non-photosynthetic tissues in the form of sucrose. The research also showed that after spraying DCPTA, the transportation of sucrose from leaves to pod and grain could be significantly improved by changing the relative gene expression of SuSy and invertase, therefore increasing the number of pods per plant, pod setting rate, and mung bean yield.

## Data Availability Statement

The datasets presented in this study can be found in online repositories. The names of the repository/repositories and accession number(s) can be found below: https://www.ncbi.nlm.nih.gov/, PRJNA717930.

## Author Contributions

YGa and YGu conceived and designed this study. YGa, XW, and XZ conducted these experiments. YGa, XL, QZ, XW, HW, BZ, TQ, and HT analyzed the data and prepared the figures and illustrations. YGa wrote the manuscript. All authors have read and approved the submission of the manuscript.

## Funding

This work was financially supported by the Heilongjiang Bayi Agricultural University Support Program (ZRCPY201920 and PTJH201905), and the Applied Technology Research and Development Program of Heilongjiang Province (GA19B104).

## Conflict of Interest

The authors declare that the research was conducted in the absence of any commercial or financial relationships that could be construed as a potential conflict of interest.

## Publisher's Note

All claims expressed in this article are solely those of the authors and do not necessarily represent those of their affiliated organizations, or those of the publisher, the editors and the reviewers. Any product that may be evaluated in this article, or claim that may be made by its manufacturer, is not guaranteed or endorsed by the publisher.
