# Supplementary material for: Exogenous DCPTA Treatment Increases Mung Bean Yield by Improving Carbon Metabolism Pathway and Up-Regulating Photosynthetic Capacity and Antioxidants
Source: Front Plant Sci. 2022 Apr 12;13:796694. doi: 10.3389/fpls.2022.796694 (PMC9039728; doi:10.3389/fpls.2022.796694)
Supplement: Supplementary file 1 [file Data_Sheet_1.doc]

| Year | pH | Organic matter g/kg | Ikali-hydro N  mg/kg | Available  P mg/kg | Available K mg/kg | Total  N g/kg | Total  P g/kg | Total  K g/kg |
| --- | --- | --- | --- | --- | --- | --- | --- | --- |
| 2020 | 7.68 | 28.6 | 176.3 | 62.1 | 213 | 1.81 | 0.69 | 0.45 |
| 2021 | 7.75 | 28.4 | 172.5 | 61.2 | 208 | 1.78 | 0.65 | 0.43 |

TABLE S1. Daily mean values of weather variables at the experimental site during each of six months of the mungbean growing season from 2020 to 2021.

| Parameter | Year | May | June | July | August | September | October |
| --- | --- | --- | --- | --- | --- | --- | --- |
| Average temperature (℃) | 2020 | 15.0 | 19.0 | 24.5 | 21.0 | 15.0 | 5.5 |
|  | 2021 | 14.5 | 20.5 | 25.0 | 20.5 | 15.5 | 6.5 |
| Precipitation (mm) | 2020 | 33.4 | 127.6 | 118.9 | 133.9 | 108.8 | 15.7 |
|  | 2021 | 37.5 | 92.8 | 222.6 | 127.0 | 66.9 | 12.1 |

TABLE S2: Raw RNA-Seq data quality analysis

|  | Total Reads | GC(%) | Q20(%) | Q30(%) | Mapped Reads | Uniq Map | Multiple Map | Only Map Plus Strand | Only Map Minus Strand | Rand Check | Insert  Size Peak |
| --- | --- | --- | --- | --- | --- | --- | --- | --- | --- | --- | --- |
| BL-1 | 64643540 | 45.68 | 97.76 | 93.63 | 60572163 (93.70%) | 56668036 (87.66%) | 3904127 (6.04%) | 30162727 (46.66%) | 30183647 (46.69%) | 0.0127 | 294 |
| BL-2 | 54297914 | 45.15 | 97.95 | 94.04 | 51847306 (95.49%) | 48392850 (89.12%) | 3454456 (6.36%) | 25722015 (47.37%) | 25742786 (47.41%) | 0.0129 | 294 |
| BL-3 | 60057910 | 45.68 | 97.89 | 93.95 | 57057259 (95.00%) | 53150621 (88.50%) | 3906638 (6.50%) | 28396390 (47.28%) | 28417643 (47.32%) | 0.0126 | 294 |
| BLCK-1 | 50896620 | 45.33 | 97.75 | 93.64 | 48322879 (94.94%) | 45381953 (89.16%) | 2940926 (5.78%) | 24053893 (47.26%) | 24082668 (47.32%) | 0.0127 | 294 |
| BLCK-2 | 51704756 | 45.26 | 97.74 | 93.62 | 49222420 (95.20%) | 46581833 (90.09%) | 2640587 (5.11%) | 24496474 (47.38%) | 24530396 (47.44%) | 0.0125 | 294 |
| BLCK-3 | 49861306 | 45.17 | 97.66 | 93.39 | 46878611 (94.02%) | 43947082 (88.14%) | 2931529 (5.88%) | 23207060 (46.54%) | 23249485 (46.63%) | 0.0129 | 308 |

TABLE S3. Raw RNA-Seq data uploaded in NCBI

| Accession | Title | Submission |
| --- | --- | --- |
| SRR14086624 | RNA-Seq of mung bean: Initial flowering stage with DCPTA. | SUB9374663 |
| SRR14086621 | RNA-Seq of mung bean: Initial flowering stage with CK. | SUB9374663 |
| SRR14086625 | RNA-Seq of mung bean: Initial flowering stage with DCPTA. | SUB9374663 |
| SRR14086622 | RNA-Seq of mung bean: Initial flowering stage with CK. | SUB9374663 |
| SRR14086623 | RNA-Seq of mung bean: Initial flowering stage with CK. | SUB9374663 |
| SRR14086626 | RNA-Seq of mung bean: Initial flowering stage with DCPTA. | SUB9374663 |
